# *Streptococcus pneumoniae* biofilm formation and dispersion during colonization and disease

**DOI:** 10.3389/fcimb.2014.00194

**Published:** 2015-01-13

**Authors:** Yashuan Chao, Laura R. Marks, Melinda M. Pettigrew, Anders P. Hakansson

**Affiliations:** ^1^Division of Experimental Infection Medicine, Department of Laboratory Medicine, Lund UniversityMalmö, Sweden; ^2^Department of Microbiology and Immunology, University at Buffalo, The State University of New YorkBuffalo, NY, USA; ^3^Department of Epidemiology and Microbial Diseases, Yale School of Public HealthNew Haven, CT, USA

**Keywords:** biofilm, colonization, streptococcus, virus, virulence

## Abstract

*Streptococcus pneumoniae* (the pneumococcus) is a common colonizer of the human nasopharynx. Despite a low rate of invasive disease, the high prevalence of colonization results in millions of infections and over one million deaths per year, mostly in individuals under the age of 5 and the elderly. Colonizing pneumococci form well-organized biofilm communities in the nasopharyngeal environment, but the specific role of biofilms and their interaction with the host during colonization and disease is not yet clear. Pneumococci in biofilms are highly resistant to antimicrobial agents and this phenotype can be recapitulated when pneumococci are grown on respiratory epithelial cells under conditions found in the nasopharyngeal environment. Pneumococcal biofilms display lower levels of virulence *in vivo* and provide an optimal environment for increased genetic exchange both *in vitro* and *in vivo*, with increased natural transformation seen during co-colonization with multiple strains. Biofilms have also been detected on mucosal surfaces during pneumonia and middle ear infection, although the role of these biofilms in the disease process is debated. Recent studies have shown that changes in the nasopharyngeal environment caused by concomitant virus infection, changes in the microflora, inflammation, or other host assaults trigger active release of pneumococci from biofilms. These dispersed bacteria have distinct phenotypic properties and transcriptional profiles different from both biofilm and broth-grown, planktonic bacteria, resulting in a significantly increased virulence *in vivo*. In this review we discuss the properties of pneumococcal biofilms, the role of biofilm formation during pneumococcal colonization, including their propensity for increased ability to exchange genetic material, as well as mechanisms involved in transition from asymptomatic biofilm colonization to dissemination and disease of otherwise sterile sites. Greater understanding of pneumococcal biofilm formation and dispersion will elucidate novel avenues to interfere with the spread of antibiotic resistance and vaccine escape, as well as novel strategies to target the mechanisms involved in induction of pneumococcal disease.

## Background

*Streptococcus pneumoniae* colonizes the upper respiratory tract in humans. Colonization occurs on the mucosal surface of the nasopharynx during childhood and persists asymptomatically in healthy individuals into adulthood (Gray et al., 1981; Hogberg et al., 2007). Pneumococcal carriage rates are greater in children compared to adults, with approximately 20–50% carriage rate in children and 5–20% in adults in higher resourced countries while even higher rates are seen in resource poor settings where up to 90% of children and over half of adults are colonized (Gray et al., 1980; Revai et al., 2008; Huang et al., 2009; Pebody et al., 2009; Mackenzie et al., 2010; Korona-Glowniak and Malm, 2012; Adegbola et al., 2014). Despite a low attack rate, transition from asymptomatic colonization to disease occurs often enough that the pneumococcus remains a leading cause of acute otitis media, pneumonia, sepsis, and meningitis globally (Sleeman et al., 2006; O'brien et al., 2009; Black et al., 2010). In 2011, *S. pneumoniae* caused an estimated 2,858,000 severe pneumonia episodes and 411,000 deaths worldwide in children under the age of 5 (Walker et al., 2013). The burden of disease is highest in resource poor settings where the lack of nutrition, antibiotics, and vaccines make the population particularly susceptible to disease.

## Properties of pneumococcal biofilms

### Introduction

Biofilms are highly-structured communities of cells that produce an extracellular matrix and adhere to abiotic or biological surfaces (Costerton et al., 1999; Donlan and Costerton, 2002; Stoodley et al., 2002). Antibacterial resistance is an inherent characteristic of biofilms and the protective biofilm matrix enables evasion of host immune responses, facilitating persistence, and dissemination of bacteria (Costerton et al., 1999; Donlan and Costerton, 2002; Chole and Faddis, 2003; Lewis, 2008; Sanchez et al., 2011b). In this context, resistance refers to an increased tolerance to antibacterials rather than a decreased susceptibility due to changes in the genome, such as mutations or obtaining antibiotic resistance genes. Pneumococcal colonization precedes disease and has been known to be more challenging to eradicate than invasive disease in patients as treatment with antimicrobial agents do not eliminate the majority of bacteria carried in the nasopharynx (Cohen et al., 1997, 1999; Dabernat et al., 1998; Dagan et al., 1998, 2001; Varon et al., 2000; Garcia-Rodriguez and Fresnadillo Martinez, 2002). Thus, a reasonable explanation for the decreased sensitivity of pneumococci to antimicrobial treatment during carriage is the formation of biofilm communities in the nasopharynx (Waite et al., 2001; Oggioni et al., 2006; Munoz-Elias et al., 2008; Trappetti et al., 2009; Sanchez et al., 2011b).

The original literature investigating pneumococcal biofilm formation *in vivo* detected biofilms during disease states such as otitis media, chronic rhinosinusitis, with some evidence for clustering of bacteria also during pneumonia (Hall-Stoodley et al., 2006; Sanderson et al., 2006; Hoa et al., 2009; Reid et al., 2009; Sanchez et al., 2011b). More recent data indicate that biofilm bacteria detected at disease sites represent asymptomatic colonization and, therefore, the presence of biofilms at sterile sites during disease presumably form a reservoir from which virulent bacteria may seed off under the right conditions, resulting in a role for biofilm bacteria in the disease process (Oggioni et al., 2006; Weimer et al., 2010; Sanchez et al., 2011b).

The vast majority of *in vitro* studies have been performed primarily on abiotic surfaces (Moscoso et al., 2006; Oggioni et al., 2006; Garcia-Castillo et al., 2007; Munoz-Elias et al., 2008; Domenech et al., 2009; Parker et al., 2009; Trappetti et al., 2009, 2011b,c; Sanchez et al., 2010, 2011a; Tapiainen et al., 2010; Camilli et al., 2011)., mimicking the classical models set up for organisms that confer problems in patients by producing biofilms on abiotic surfaces associated with medical devices. The extent of relevance these *in vitro* studies have *in vivo* is unclear as most of the biofilm formation experiments were conducted over short periods of time on abiotic surfaces that, as far as we know, are not major natural environments for the pneumococcal life cycle. For the same reason, *in vitro* studies on abiotic surfaces conducted for longer periods of time have unclear *in vivo* implications (Allegrucci et al., 2006; Allegrucci and Sauer, 2007; Vandevelde et al., 2014). Additional studies utilizing clinical isolates to study biofilms with longer biofilm formation times have been unable to show any association between the ability to produce *in vitro* biofilms on abiotic surfaces and *in vivo* virulence (Lizcano et al., 2010; Tapiainen et al., 2010). Furthermore, controversy exists in the literature regarding the correlation between biofilms grown *in vitro* on abiotic surfaces and their infectivity *in vivo* where investigators have suggested that biofilm bacteria are more likely (Trappetti et al., 2011b) or less likely (Sanchez et al., 2011b) to cause invasive disease. Our data at this point support the notion that biofilm bacteria are less virulent in invasive disease models (Marks et al., 2013). The virulence of biofilm bacteria will be covered in more depth in a separate review in this topic series by Orihuela et al. (Cross-reference to Orihuela review) (Gilley and Orihuela, 2014).

While these studies have been essential in building our understanding of pneumococcal accretion and biofilm formation, studies with more complex model systems that include physiological conditions and components modeling host–pneumococcal interactions have only recently shed more light on the phenotype of biofilm bacteria. In a study by Parker et al. bacteria recovered after adhering to epithelial cells had an increased ability to form biofilms on abiotic surfaces compared to bacteria with no previous exposure to epithelial cells (Parker et al., 2009). Also, Sanchez et al. found that biofilm bacteria grown on abiotic surfaces adhered better to epithelial cells than planktonic, broth grown bacteria (Sanchez et al., 2011b). These two studies, supported by studies in other human pathogens (Konkel et al., 1997; Sulaeman et al., 2012), demonstrate a relationship between epithelial cell adherence and biofilm formation, however, the studies have not investigated the role of this relationship during pneumococcal colonization.

### Formation of well-organized and structured biofilms during nasopharyngeal colonization

**Figure 1:**
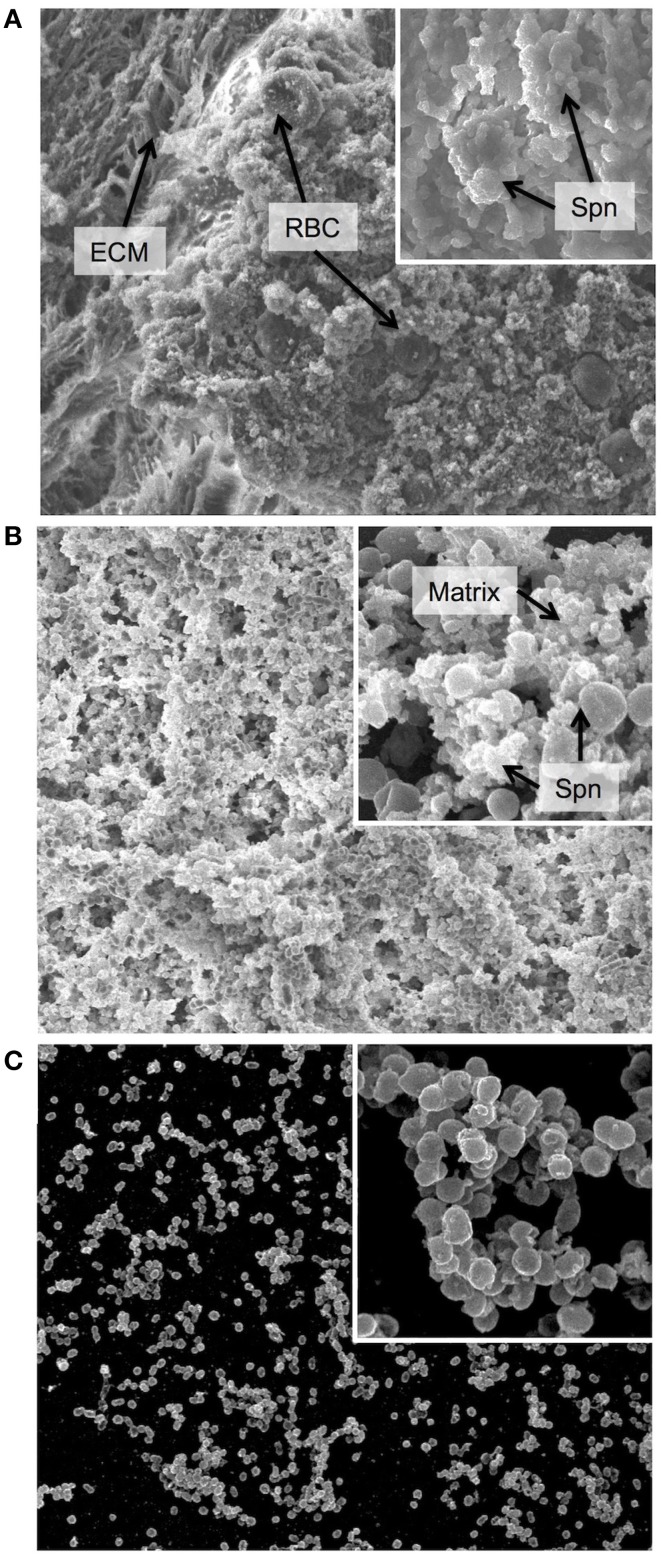
**Biofilm morphology**. Scanning electron micrographs of biofilm communities formed **(A)** on the epithelial mucosa *in vivo*, **(B)** on epithelial cells grown *in vitro* and **(C)** on a glass substratum *in vitro*. The major image in each panel shows the biofilm at 2000x magnification and the insert in the upper right corner shows an increased magnification of 10,000x. ECM = extracellular matrix, used here instead of the more conventional EPS (extracellular polymeric substance) as secretion of specific polymeric substances have not yet been identified or characterized in *Streptococcus pneumoniae*, and the matrix is not well defined. RBC = red blood cell, Spn = *Streptococcus pneumoniae*, Matrix = biofilm matrix composed of extracellular substances and cellular debris. In general, the *in vivo* biofilms display a high degree of matrix formation that originated primarily from lysed bacterial cells and consists of cellular debris and DNA. Biofilms from *in vitro* cultures on epithelial cells show less encapsulation in matrix and more naked bacterial cells. However, biofilms formed on glass are much less developed with less biomass and almost no matrix formation.

Researchers have speculated that pneumococci form biofilms in the nasopharynx *in vivo* (Waite et al., 2001; Oggioni et al., 2006; Munoz-Elias et al., 2008; Trappetti et al., 2009; Sanchez et al., 2011b). Recently, Marks et al. showed for the first time that pneumococci form highly structured biofilms during colonization of the murine nasopharynx (Marks et al., 2012a) BALB/c mice were inoculated intranasally with the pneumococcal strain EF3030, a clinical isolate known to be non-invasive and an efficient colonizer in murine models (Balachandran et al., 2002; Palaniappan et al., 2005; Shah et al., 2009). After 48 h, the pneumococcal carriage was 5 × 10^6^ organisms per nasopharyngeal tissue, similar to other studies using EF3030 (Briles et al., 2003; Palaniappan et al., 2005; Shah et al., 2009). Scanning electron microscopy (SEM) images of excised nasopharyngeal tissue showed colonization on ciliated epithelium with a higher bacterial burden and increased biofilm density in posterior sections of the nasopharynx compared with the anterior sections (Figure 1A) (Marks et al., 2012a). In the anterior region we found pneumococcal single cells or diplococci scattered in the tissue. In contrast, aggregated and interconnected cells with tower and filamentous structures covered in extracellular matrix were observed in the posterior region layered on top of the ciliated epithelium. Other bacterial species were not identified in nasal tissues of the infected mice and no bacterial growth was observed in uninfected mice. These data have been confirmed by the Orihuela group that found biofilm formation on nasal septa during colonization of the murine nasopharynx (Blanchette-Cain et al., 2013). In their study, they found that biofilm formation during colonization required the CiaR/H two component system and that PsrP and SpxB had a major impact on bacterial aggregation, whereas CbpA, LuxS, and LytA had only modest effects.

### Biofilms display increased resistance to antimicrobial agents

Formation of bacterial biofilms confers greatly increased resistance to antimicrobial agents (Costerton et al., 1999; Donlan and Costerton, 2002; Chole and Faddis, 2003; Lewis, 2008; Sanchez et al., 2011b). The biofilm structure functions as a shield and protects the bacteria from the antimicrobials. Increased resistance, in this sense, has partly been attributed to a somewhat lower penetration of antibiotics into the biofilm structure but is probably equally or more associated with an adaptive phenotype shift of the biofilm bacteria (De Kievit et al., 2001; Drenkard and Ausubel, 2002; Nguyen et al., 2011; De La Fuente-Nunez et al., 2013). However, resistance to antimicrobial agents can be discussed as tolerance to antibiotics or as a result of acquired genes due to genetic exchange as will be discussed below in Section Role of Biofilm Formation during Pneumococcal Colonization. Biofilm bacteria constitute a heterogeneous population, with many bacteria in a more sessile state, having the “persister” phenotype described by Lewis (2008) or expressing other adaptive changes to resist environmental stressors (De La Fuente-Nunez et al., 2013).

This appears true also in pneumococcal biofilms. In our studies, treatment with gentamicin was used to test the functional and structural organization of the biofilm as the antibiotic is bactericidal against planktonic bacteria but does not penetrate well-organized biofilms effectively (Carmen et al., 2004; Abdi-Ali et al., 2006; Bartoszewicz et al., 2007). We also examined the effect of penicillin, a commonly used antibiotic, to provide clinically relevant results. Pneumococci closely associated with the murine nasopharyngeal tissue are highly resistant to gentamicin and penicillin while loosely associated bacteria are eradicated at a much lower concentration of antibiotics (Marks et al., 2012a), supporting the previous findings that showed a higher persistence of colonizing bacteria than those causing disease (Cohen et al., 1997, 1999; Dabernat et al., 1998; Dagan et al., 1998, 2001; Varon et al., 2000; Garcia-Rodriguez and Fresnadillo Martinez, 2002) and that biofilms are inherently more resistant to antibacterial agents (Costerton et al., 1999; Donlan and Costerton, 2002; Chole and Faddis, 2003; Lewis, 2008; Sanchez et al., 2011b). Enhanced resistance to aminoglycoside and beta-lactam antibiotics may also result from oxygen limitation as shown in *Escherichia coli* (Tresse et al., 1995, 1997). Altogether, these data suggest that biofilm formation during colonization may provide one mechanism that the pneumococci utilize to persist during antibiotic exposure in the human host.

### Models to study bacterial–host interactions *in vitro*

A challenge in any study of host–bacterial interactions is to recapitulate *in vivo* findings using *in vitro* models. As described above (Section Introduction), the majority of work with pneumococcal biofilms has relied on *in vitro* model systems in which host-specific factors have not been included or examined. The nasopharyngeal environment contains a mucosal surface of respiratory epithelium and their secretions. This environment provides challenges to the bacterial organisms with low nutrient availability and also a lower temperature than the remaining body (approximately 32–34°C rather than 37°C) (Keck et al., 2000; Sahin-Yilmaz and Naclerio, 2011).

Our group has developed an *in vitro* model to simulate the upper respiratory tract, the site of pneumococcal colonization (Marks et al., 2012a). Biofilms grown on abiotic surfaces were delayed in growth and had lower biomass and lacked structures seen in biofilms grown on epithelial cells or *in vivo* during nasopharyngeal colonization (Figure 1), suggesting that interactions with epithelial cells play an important role in biofilm formation (Parker et al., 2009; Sanchez et al., 2011b). Biofilms grown on live or fixed epithelial substrata formed complex biofilms with high biomass, similar matrix formation, general architecture and organization, and functional characteristics (e.g., antibiotic resistance) as biofilms formed *in vivo* in the mouse nasopharynx, thereby providing a suitable *in vitro* surrogate model for biofilm formation *in vivo* (Figure 1B). Both healthy human respiratory epithelial cells grown and differentiated in an air-liquid interphase and bronchial carcinoma cells support robust biofilm development (Figure 1B). This was not observed when biofilms were grown under the same conditions on plastic surfaces (Figure 1C). Moreover, the differences in structure and maturation also impacted on levels of gentamicin resistance; with biofilms grown on abiotic surfaces having decreased antibiotic resistance compared to biofilms grown on epithelial substrata. These phenotypic differences may indicate that abiotic surfaces lack important *in vivo* features to support optimal biofilm formation. There is one other group that has used epithelial cells as a substratum for biofilm formation. Vidal et al. used both paraformaldehyde-fixed HEp-2 epithelial and A549 lung carcinoma cells to produce static biofilms *in vitro* and also produced biofilms in a flow chamber bioreactor (Vidal et al., 2013). Consistent with our studies, the biomass of the biofilms was significantly higher in the presence of epithelial cells, and in their system more biomass was associated with the lung cells than the HEp-2 cells. Using this system, the authors were able to verify their earlier studies showing that both competence induction and autoinducer production is important for early biofilm formation (Vidal et al., 2011, 2013).

Environmental factors such as temperature and nutrient availability also impact biofilm formation. An environment of 34°C results in more dense and functional biofilms than biofilms formed at 37°C, measured both through morphology in SEM and by resistance to antimicrobial agents. These data suggest that the physiological temperature of the nasopharyngeal niche provides more optimal conditions to support biofilm formation. Finally, nutrient availability also impacts biofilm formation as nutrient-rich media did not support biofilm development as well as media containing fewer nutrients (Marks et al., 2012a,b).

### Correlation between biofilm formation and the ability for *in vivo* colonization

To validate the degree of correlation between our *in vitro* biofilm model and *in vivo* colonization, we compared the functional biofilm formation of pneumococcal strains with the bacterial burden during colonization of the same strains. The ability to form biofilms on epithelial cells directly correlated with the ability to colonize the murine nasopharynx (Marks et al., 2012a). Specifically, clinical isolates (EF3030 and BG8826) known to be effective colonizers of the murine nasopharynx (Lipsitch et al., 2000; Briles et al., 2003) formed more developed biofilms with higher biomass and biofilm-specific antibacterial resistance on epithelial cells than more invasive strains that are known to colonize the murine nasopharynx less effectively (D39, WU2, and SP670) (Benton et al., 1995; Briles et al., 2003; Mizrachi-Nebenzahl et al., 2003; Orihuela et al., 2003). Moreover, colonization-deficient strains in the D39 background that lacked virulence-associated factors, such as autolysin, pneumolysin, and PspC formed less structured, more antibiotic-sensitive biofilms, whereas pneumococci lacking PspA that is not associated with early colonization showed normal biofilm formation (Marks et al., 2012a). These differences were not observed in biofilms formed on abiotic surfaces.

## Role of biofilm formation during pneumococcal colonization

Forming biofilms during colonization may serve several purposes for pneumococci. The biofilm provides a protective environment, in which the bacteria can adapt to coexist with the host by down-regulating factors involved in inducing inflammation in favor of factors used to scavenge nutrients from the harsh environment in the nasopharynx. This will be discussed more below in Section Distinct Phenotypic Properties of Dispersed Pneumococci. Another benefit of biofilm communities is the closeness of bacterial cells to each other as well as the proximity to DNA that makes up part of the extracellular matrix, providing an excellent environment to exchange genetic material to promote survival and adaptation to the host environment.

Pneumococci are highly competent organisms and their genome sequences show extensive signs of horizontal transfer of genetic material. The mechanism of competence initiation, DNA uptake and integration has been well studied in *S. pneumoniae* (Johnsborg and Havarstein, 2009) since the first observation of natural genetic transformation by Griffith (1928). Horizontal gene transfer is important for adapting to environmental stresses (Stewart and Carlson, 1986; Johnsborg et al., 2007), as it enables the acquisition of novel traits and the spread of antibiotic resistance (Majewski et al., 2000; Hakenbeck et al., 2001; Claverys et al., 2007). Previous studies have reported low levels of spontaneous DNA uptake and transformation in *S. pneumoniae* strains *in vivo* (Griffith, 1928; Ottolenghi and Macleod, 1963; Conant and Sawyer, 1967; Zhu and Lau, 2011). However, these *in vivo* studies were performed in the context of sepsis or other disease states where the level of biofilm formation is low. Additionally, most of the studies investigating natural transformation have used hypercompetent lab strains derived from Avery's experiments (Avery et al., 1944) or clinical isolates that require the addition of synthetic competence-stimulating peptide (CSP) (Pozzi et al., 1996; Wei and Havarstein, 2012).

Epidemiological studies suggest that colonizing bacteria rather than bacteria from invasive disease are the source of horizontal transfer or spread of antibiotic resistance between strains (Christenson et al., 1997; Nasrin et al., 1999; Ronchetti et al., 1999; Domenech et al., 2009) and that resistance selection occurs mainly in pneumococci colonizing young children, an age-group that has high carriage rates and exposure to antibiotics that consequently favor the selection of drug-resistance (Duchin et al., 1995; Samore et al., 2001; Brugger et al., 2009). This is supported by studies suggesting that natural transformation in the nasopharynx is facilitated by co-colonization of multiple pneumococcal strains (Donkor et al., 2011; Leung et al., 2011). In addition, pneumococcal biofilm formation during colonization of the nasopharynx has been shown to up-regulate competence genes (Oggioni et al., 2006; Trappetti et al., 2011a).

### Increased natural transformation in bacteria during colonization compared with sepsis

**Figure 2:**
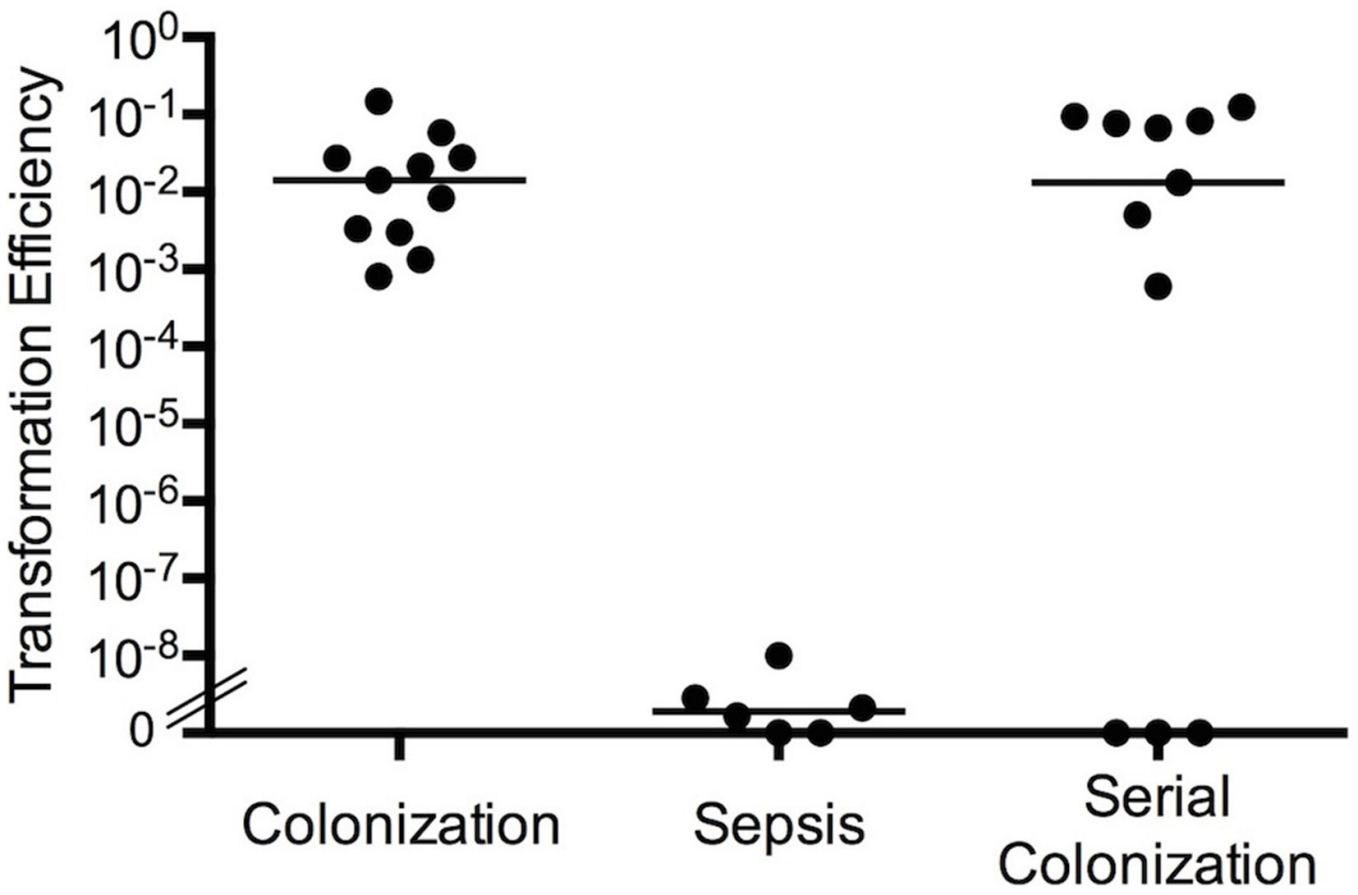
**Transformation efficiency in biofilm cultures and during nasopharyngeal colonization**. Transformation efficiency of antibiotic resistance elements between *S. pneumoniae* strain SP670 (Pen^R^) and D39-C08P2 (Erm^R^). Mice were co-colonized with both strains at the same time, colonized sequentially with one strain added to the nares of mice 48 h prior to the second strain, or both strains were used for co-infection in a septicemia model in mice. Transformation efficiency was measured as the number of double-antibiotic resistant colonies divided by total recovery of bacteria from each condition.

To further study the *in vivo* signals and host conditions involved in increased natural transformation between strains *in vivo*, we performed experiments using our *in vitro* biofilm model as well as investigated transformation during colonization. When BALB/c mice were inoculated intranasally or intraperitoneally with equal numbers of *S. pneumoniae* strains SP670 (a clinical penicillin-resistant strain) and D39-C08P2 (a laboratory strain with an erythromycin cassette inserted downstream of the dihydrolipoamide dehydrogenase gene), natural transformation only occurred in bacteria colonizing the nasopharynx (Marks et al., 2012b). The transformation efficiency (the ratio of the number of double-resistant colonies over the total recovered population) indicated that colonizing bacteria in the murine nasopharynx showed a surprisingly high level of natural transformation (efficiency of ~1 × 10^−2^) whereas the natural transformation efficiency during sepsis was very low and similar to what has been presented in the literature (efficiency of 3 × 10^−9^) (Figure 2). Thus, the transformation efficiency during colonization was approximately 10^7^-fold higher than during sepsis. Also, sequential nasopharyngeal colonization, where one strain was inoculated and left to colonize the animals for 48 h before the other strain was added intranasally, had similar transformation efficiencies as when the strains were inoculated simultaneously (Figure 2). This model better mimics the natural, sequential acquisition of strains and the combined data is in agreement with epidemiological studies suggesting that colonizing bacteria are the predominant source of horizontal transfer of genes between strains (Christenson et al., 1997; Nasrin et al., 1999; Ronchetti et al., 1999; Doit et al., 2000).

The *in vivo* data could be corroborated *in vitro* using biofilms grown on epithelial cells at 34°C (Marks et al., 2012b). Seeding epithelial cells with equal numbers of the two antibiotic-resistant strains resulted in high numbers of double-resistant organisms. The highest transformation frequency was observed between 48 and 72 h after inoculation, which corresponded directly to the time points when the average competence gene expression in the biofilm population was highest. As our previous work demonstrated that pneumococcal biofilm formation occurs during nasopharyngeal colonization (Marks et al., 2012a) and transformation efficiency is increased in co-colonization or serial colonization compared to sepsis (Marks et al., 2012b), this suggests that biofilm formation plays a role in the increased genetic exchange seen during colonization. This is in agreement with another study showing more efficient gene transfer among streptococci in early biofilm structures (Wei and Havarstein, 2012). Moreover, several studies show that the matrix of most biofilms contains high concentrations of DNA that originate from lysis of bacterial cells in the biofilm (Thomas et al., 2009; Kiedrowski et al., 2011; Liu and Burne, 2011; Montanaro et al., 2011). Lysis can be obtained through autolysis but may also result from phage-mediated bacterial host lysis, enhancing pneumococcal biofilm development as measured by biomass and cell viability (Carrolo et al., 2010). In addition, it is known that the pneumococcal process of fratricide releases DNA from a subfraction of the population by triggered cell lysis due to competence development (Steinmoen et al., 2002). Biofilm growth may therefore provide an optimal environment for genetic exchange, further suggested by data where encapsulated strains that show no natural transformation *in vitro* during growth in broth can integrate resistance cassettes during biofilm growth with a transformation efficiency of 10^−3^ to 10^−4^ after addition of extracellular chromosomal DNA (1 μg/mL) without exogenous addition of CSP or antibiotic pressure (Marks et al., 2012b).

### Mechanisms of increased natural transformation in biofilms

#### Induced competence

Biofilms have been shown to upregulate competence genes compared with broth-grown bacteria (Oggioni et al., 2006; Trappetti et al., 2011a). As mentioned above, it appears that competence is continuously upregulated during biofilm growth on epithelial cells. This does not necessarily mean that the total population of the biofilm is competent all the time. Rather, biofilms are heterogeneous and dynamic populations, suggesting that although the average competence gene expression in a biofilm is continuously high, this most likely reflects up- and down-regulation of competence in subpopulations within the biofilms. Under optimal biofilm-growth conditions, the constant presence of exogenous CSP did not increase the already high transformation efficiencies in biofilms (Marks et al., 2012b). However, when biofilms were formed under sub-optimal conditions such as in the presence of rich media (Todd-Hewitt medium containing yeast extract, THY) or at 37°C rather than 34°C, or on abiotic surfaces, addition of CSP significantly improved both biofilm formation and transformation efficiencies. The role of competence induction in biofilm formation is supported by several investigators that have shown that inclusion of competence stimulating peptide increases the biomass of biofilms (Oggioni et al., 2006; Trappetti et al., 2011c). These differences were not seen in assays testing natural transformation during planktonic growth.

#### Capsule down-regulation

Capsule expression is affected by environmental factors (Selinger and Reed, 1979; Kim and Weiser, 1998; Weiser et al., 2001; Hammerschmidt et al., 2005) and phenotypic variation can occur in the transition from nasopharyngeal carriage to invasive disease (Waite et al., 2003). Transparent variants with thinner capsule are predominantly found during initial colonization while opaque strains with thicker capsule are found during invasive disease (Weiser et al., 1994; Cundell et al., 1995; Kim and Weiser, 1998; Kim et al., 1999). Increased capsule expression results in decreased transformation efficiency (Ravin, 1959) and only unencapsulated strains have been found to be naturally transformable in broth. We have found that the capsule locus is downregulated in biofilms compared with bacteria grown in broth (Marks et al., 2012b). Similar results have been presented in another study where biofilms grown on an abiotic substratum were compared with planktonic cultures (Hall-Stoodley et al., 2008). Altogether, these data suggest that capsule down-regulation during biofilm formation and colonization result in the increased transformation efficiency seen during biofilm growth.

#### Epithelial interactions

As the down-regulation of capsule was more pronounced when grown on epithelial cells than when biofilms formed on abiotic surfaces (Hall-Stoodley et al., 2008; Marks et al., 2012b), epithelial cells may play a major role in this regard. This is supported by a study from Hammerschmidt's laboratory showing that pneumococci downregulate their capsule when adhering to epithelial cells (Hammerschmidt et al., 2005). In our dual-strain biofilm studies, biofilms formed both on prefixed epithelial cells or glass displayed an elevated level of transformation efficiency (Marks et al., 2012b). However, the presence of a prefixed epithelial substratum resulted in a higher transformation efficiency than observed on glass. This further indicates the significance of bacteria–host interactions for optimal biofilm formation, which in turn potentiates effective transformation.

#### Nutrient availability

Other studies have shown that ion and nutrient concentrations play a role during transformation of planktonic pneumococcal cultures (Lacks and Greenberg, 1973; Chen and Morrison, 1987; Trombe, 1993). As previously mentioned, pneumococci grown in varying nutrient conditions show different abilities to form biofilms that correspond with their ability to promote transformation. Limited nutrients seems to be important for optimal biofilm formation as rich, complex media (THY) resulted in poor biofilm formation with low transformation efficiencies compared to biofilms formed in chemically defined media (CDM) (Marks et al., 2012b). Therefore, the nutrient environment seems to influence genetic exchange through its initial effects on biofilm formation.

#### Nasopharyngeal temperature

Temperature has been found to modulate competence development in pneumococci cultures (Lacks and Greenberg, 1973; Steinmoen et al., 2003) and studies of the role of temperature on transformation efficiency in broth cultures have indicated that transformation efficiency peaks around 32–34°C and decreases with increasing and decreasing temperatures (Hotchkiss, 1957). During colonization of the upper respiratory tract, pneumococci are exposed to temperatures of about 34°C, which are closer to the optimal temperature for transformation than is body temperature. Dual-strain biofilms consisting of strains with separate antibiotic-resistance markers were able to form at 37°C although with lower biomass than seen at 34°C (Marks et al., 2012b). However, temperature was extremely important for natural transformation in biofilms as no transformants could be recovered at the higher temperature while high transformation efficiency was seen at 34°C. For one strain pair, biofilms did not form at 37°C, whereas at 34°C this strain pair was able to form biofilms with high transformation efficiency. Similar results were seen in single-strain biofilms with the addition of exogenous DNA, although differences between the two temperatures were not as distinct. However, biofilms with comparable biomasses had similar transformation efficiencies.

Biofilm formation occurs during colonization of the nasopharynx by *S. pneumoniae*. This niche has specific growth conditions, including epithelial interactions, nutrient availability, and temperature that are optimal for the formation of biofilms. In contrast to planktonic growth, downregulation of capsule and induction of competence occurs in biofilms. Together these environmental factors are important both for pneumococcal biofilm formation *in vitro* and during nasopharyngeal colonization *in vivo*, as well as for the ensuing increased genetic exchange and natural transformation.

### Population dynamics and increased fitness

Natural genetic transformation in the biofilm environment serves to increase the adaptation of the bacteria to a changing host environment and thus increases the fitness of the organism. Our mechanistic studies of biofilm-associated transformation revealed a separate mechanism whereby biofilms can promote fitness. We used a PspC- and PspA-negative strain (TRE121; erythromycin-resistant and tetracycline resistant) to investigate whether the *pspC* locus required for colonization (Balachandran et al., 2002) could be repaired if grown in the presence of the wild-type strain. Repair could then be observed by detecting only erythromycin-resistant bacteria carrying the PspA mutation as PspA is not required for early colonization. Mice inoculated with *pspA/pspC* null pneumococci alone were rapidly cleared. However, intranasal inoculation of a mixture of TRE121 and D39 pneumococci resulted in a population with the *pspC* gene (erythromycin resistant and tetracycline sensitive) repaired genetically and functionally. These studies supported that natural transformation during co-colonization can improve fitness by expanding the gene pool available for adaptation to the host environment (Marks et al., 2012b).

However, in the same experiment, where TRE121 were co-colonized with wild type D39, we were also able to isolate the original PspC- and PspA-negative mutant, that when colonized alone was rapidly cleared. When performing our transformation experiments there seemed to be a trend, although not statistically significant, of increased biomass in multi-strain biofilms than single-species biofilms with equal inocula. Further investigations of dual-strain biofilms *in vitro* revealed that poor biofilm formers showed an increased biomass in the presence of good biofilm-forming strains. This increased fitness was not directly associated with acquisition of genetic factors as strains had the same colonization efficiency before and after co-colonization experiments. This fitness increase was also observed in co-colonization experiments with unencapsulated and encapsulated strains. In addition to providing an optimal environment for genetic exchange, co-colonization may also provide a haven for poorly colonizing strains when an effectively colonizing strain is also present. These data are supported by epidemiological studies showing the detection of rare serotypes or non-typeable pneumococci significantly more often in individuals colonized with multiple strains than with single strains (Brugger et al., 2010).

## Mechanism of transition from asymptomatic biofilm colonization to dissemination and disease

Pneumococcal colonization of the nasopharynx is frequent in children (20–90%, with the higher numbers observed in resource-poor settings (Hill et al., 2006; Coles et al., 2011; Kwambana et al., 2011; Abdullahi et al., 2012)), and decreases, although not completely, in adulthood. Colonization always precedes infection (Kadioglu et al., 2008), however, the mechanism involved in the transition from biofilm colonization to disease is not entirely clear. Numerous studies have suggested that pneumococcal infection is associated with preceding or concomitant virus infections (Henderson et al., 1982; Chonmaitree et al., 1986, 2012; Kim et al., 1996; McCullers, 2006; Bakaletz, 2010; Pettigrew et al., 2011; Launes et al., 2012; Chertow and Memoli, 2013; Short et al., 2013) while other studies suggest that virus infections increase bacterial growth or dissociation from the nasopharyngeal tissue (Diavatopoulos et al., 2010; Vu et al., 2011). For example, Influenza A virus (IAV) is associated with an increased susceptibility to pneumococcal pneumonia (Morens et al., 2008; Shrestha et al., 2013; McCullers, 2014). IAV pathogenesis involves invasion and killing of respiratory epithelial cells, increased bacterial adhesion receptors in the respiratory niche, and suppression of immune responses to *S. pneumoniae* (McCullers and Bartmess, 2003; Sun and Metzger, 2008; Koppe et al., 2012; McCullers, 2014). Furthermore, IAV infection is associated with increased spread between infant mice, suggesting a role for IAV in release of pneumococci from biofilm colonization in order to spread between individuals (Diavatopoulos et al., 2010). While virus infection and host signals seem to influence nasopharyngeal biofilm communities, the exact mechanism(s) whereby transition from asymptomatic colonization to disease occur have been less studied.

### Influenza a virus infection promote biofilm dispersal *in vitro* and transition to disease *in vivo*

#### *In vitro* biofilm dispersal

Using IAV as a model system we have recently attempted to address the factors associated with disruption of biofilm colonization in the nasopharynx. Previous models with human respiratory epithelial cells (HRECs) have been limited by short coexistence times between the bacteria and epithelial cells (Hakansson et al., 1996; Marks et al., 2012a; Vidal et al., 2013). We recently developed a static biofilm model with live cultures of HRECs that survived with biofilm bacteria for up to 72 h and permitted the study of the role of virus infection on biofilm integrity (Marks et al., 2013).

Pneumococcal biofilms that were first formed on fixed HRECs were moved to live cells and were allowed to reestablish a biofilm for 24 h. At this time, IAV infection of the epithelial cells were performed. At 24 h after IAV infection, the total bacterial load did not differ between cells infected or not infected with virus. However, about 10-fold more bacteria were found in the supernatant than in the biofilm communities associated with the virus-infected epithelium. The increased bacterial numbers in the supernatant was found for several pneumococcal strains and was not associated with detachment of cells.

#### *In vivo* transition to disease

As IAV infection of epithelial cells *in vitro* results in release of bacteria from biofilms, we investigated the impact of IAV infection on pneumococcal colonization *in vivo* (Marks et al., 2013). Mice were colonized intranasally with EF3030 or D39 pneumococci for 48 h, the mice were then inoculated with IAV, and bacterial burden in various tissues was measured at days 1 and 5 post infection. EF3030 biofilms maintained stable colonization of the nasopharynx over 5 days, with a slightly higher level of colonization in the IAV-infected population. The increased colonization after IAV infection has been observed in earlier studies (Hirano et al., 1999; Tong et al., 2000; Garcia-Rodriguez and Fresnadillo Martinez, 2002; Diavatopoulos et al., 2010) and was recently shown to rely on increased growth of pneumococci due to increased availability of sialic acid from IAV neuraminidase activity (Siegel et al., 2014). Associated with the increased colonization, dissemination into the lungs and the middle ear of EF3030 increased over time in the presence of IAV. For D39, colonization was higher in the IAV population but total colonization decreased over time and, although IAV caused dissemination both into the lungs and the middle ear, the initial dissemination and bacterial burden decreased over time. These results showed that IAV infection could cause active egress of bacteria from biofilms and that those bacteria could disseminate in the host to otherwise sterile sites where they caused infection.

### The role of IAV-induced changes in the host environment on biofilm dispersal and transition to disease

#### *In vitro* biofilm dispersal

Upon IAV infection, a 10-fold increased ATP concentration was detected in the biofilm supernatant at 24 h, which was similar both in fold-change and in levels detected in the nasopharyngeal lavage fluid from mice infected with IAV for 24 h. Extracellular ATP as well as the recently described IAV-induced sympato-mimetic response resulting in release of norepinephrine (NE) in the nasopharyngeal secretions constitutes well described “danger signals” potentially recognized by bacterial cells (Grebe et al., 2010; Xi and Wu, 2010). Additionally, symptomatic IAV infection is likely to cause increased or changed nutrient availability in the nasopharynx and is usually accompanied with fever, two additional factors that were shown above to have a negative impact on biofilm formation and genetic transformation (Marks et al., 2012b). To avoid host cell-mediated responses, potential host agents induced by virus infection were applied exogenously to biofilms formed on fixed epithelia. The addition of NE, ATP, glucose, or HREC lysate induced dispersal of bacteria from tissue-attached biofilm communities into the supernatant, predominantly in the form of diplococci, a pneumococcal morphology previously found in the bloodstream or sputum of patients and animals (Tomasz et al., 1964). Exposure to febrile-range hyperthermia (FRH) at an elevated temperature of 38.5°C showed similar results and the combination of 38.5°C and HREC cell lysates showed additive effects, suggesting that during IAV infection the combined effect of the changing host environment likely produced the dispersal of bacteria from biofilms.

#### *In vivo* transition to disease

**Figure 3:**
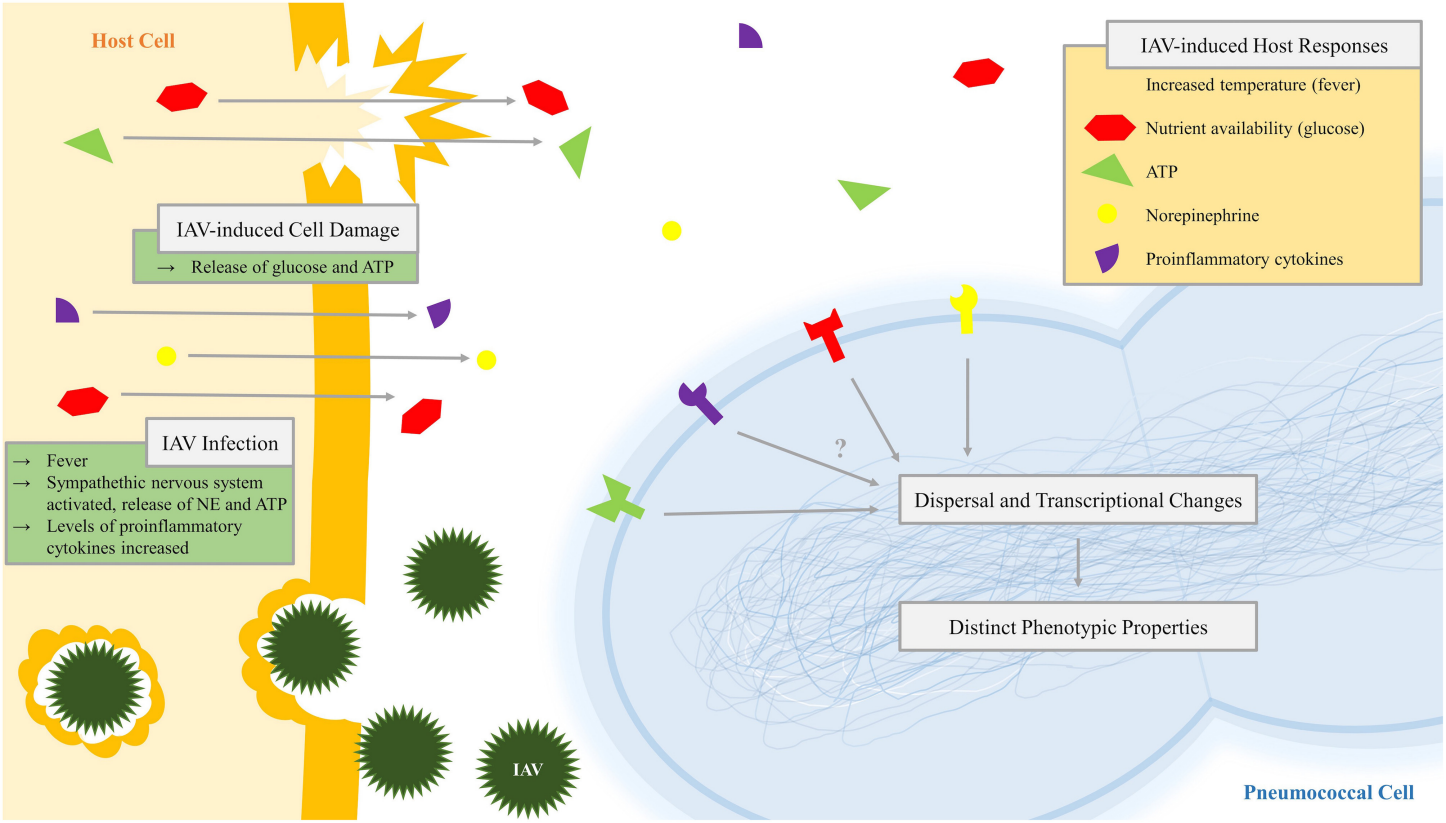
**Diagram of pneumococcal recognition of influenza-induced host responses leading to dispersal and transcriptional change**. Infection with influenza A virus (IAV), which may include virus-induced host cell damage, leads to changes in the pneumococcal niche environment including virus-induced host responses such as increased temperature (fever), nutrient availability (glucose), extracytoplasmic ATP, norepinephrine, and proinflammatory cytokines. Pneumococci recognize these inter-kingdom signals by an unknown mechanism, leading to dispersal and changes in the transcriptome and resulting in pneumococcal populations with distinct phenotypic properties.

Similar to our *in vitro* studies above, host signals (ATP, NE, glucose, and FRH) resulted in dispersion of EF3030 and D39 from the nasopharynx and caused dissemination of pneumococci into the lungs or middle ear. These data show that host-derived inter-kingdom signals alone or in conjunction with IAV infection cause active dispersal of bacteria from the biofilm, which can subsequently disseminate to normally sterile sites and cause symptomatic infection (Marks et al., 2013). The mechanisms of IAV-induced transition from colonization to infection are depicted in Figure 3. The recognition of host factors by bacteria is an underexplored area (Hughes and Sperandio, 2008; Pacheco and Sperandio, 2009) where the main examples of how bacteria recognize the host environment is associated with sensory membrane kinases of two component systems and with a few examples known where bacteria can recognize and bind host-specific molecules such as cytokines. Future studies focused on understanding how bacteria recognize changes in their environment will be of great interest to understand host–pathogen interaction both during colonization and infection. A better knowledge of these mechanisms may help provide novel strategies to avoid transition to infection.

## Distinct phenotypic properties of dispersed pneumococci

### *In vivo* phenotype of dispersed, planktonic, and biofilm populations

It has been shown that biofilm bacteria display lower virulence *in vivo* than broth-grown bacteria (Blanchette-Cain et al., 2013; Qin et al., 2013). However, the specific virulence phenotype of bacteria that are actively released from biofilms in response to a changing host environment (increased temperature, virus infection, etc.) has not been well described. Using an *in vivo* murine model of colonization and dissemination, we were able to confirm that actively-dispersed bacteria have a distinct phenotype from biofilm or planktonic, broth-grown bacteria (Marks et al., 2013). In general, the dispersed pneumococci were able to colonize the nasopharynx as well as the other populations, but disseminated into the lungs and middle ear at a higher degree than both planktonic, broth-grown bacteria and biofilm bacteria. This is in agreement with a previous study where opaque, broth-grown bacteria were able to translocate to the lungs and brain of mice while transparent, biofilm-derived bacteria remained in the nasopharynx (Trappetti et al., 2011b).

Actively-dispersed bacteria also induced a higher level of inflammation. Histological examination 7 days after colonization showed that mouse tissue infected with dispersed bacteria resulted in denudation of the epithelium, later supported in other studies (Blanchette-Cain et al., 2013), and had the presence of pronounced leukocyte infiltrates in the lungs and middle ear cavity. In agreement with the low bacterial load found in the tissues infected with biofilm-grown bacteria, there were no inflammatory infiltrates present. However, the nasal epithelium had shorter cilia compared to mock-infected mice. Mice inoculated with planktonic, broth-grown bacteria displayed a mixed phenotype, showing areas of epithelial denudation and some inflammation in the middle ear and lungs. Histological results were very similar between D39 and EF3030 pneumococci with the exception that no D39 bacteria were isolated from the middle ear.

After direct aspiration of bacterial populations into the lungs of mice, biofilm bacteria were cleared over time, induced minimal inflammation, and did not disseminate into the bloodstream. In contrast, temperature-dispersed biofilm bacteria caused high levels of bacterial burden in the lungs with three out of six mice challenged with EF3030 and all of the mice challenged with D39 showing pneumococcal dissemination into the bloodstream. Histological analysis of the lungs infected with dispersed bacteria showed a dense leukocyte infiltrate with hemorrhagic lesions, while planktonic, broth-growth bacteria showed a moderate bacterial burden in the lungs, resulting in moderate inflammation.

**Figure 4:**
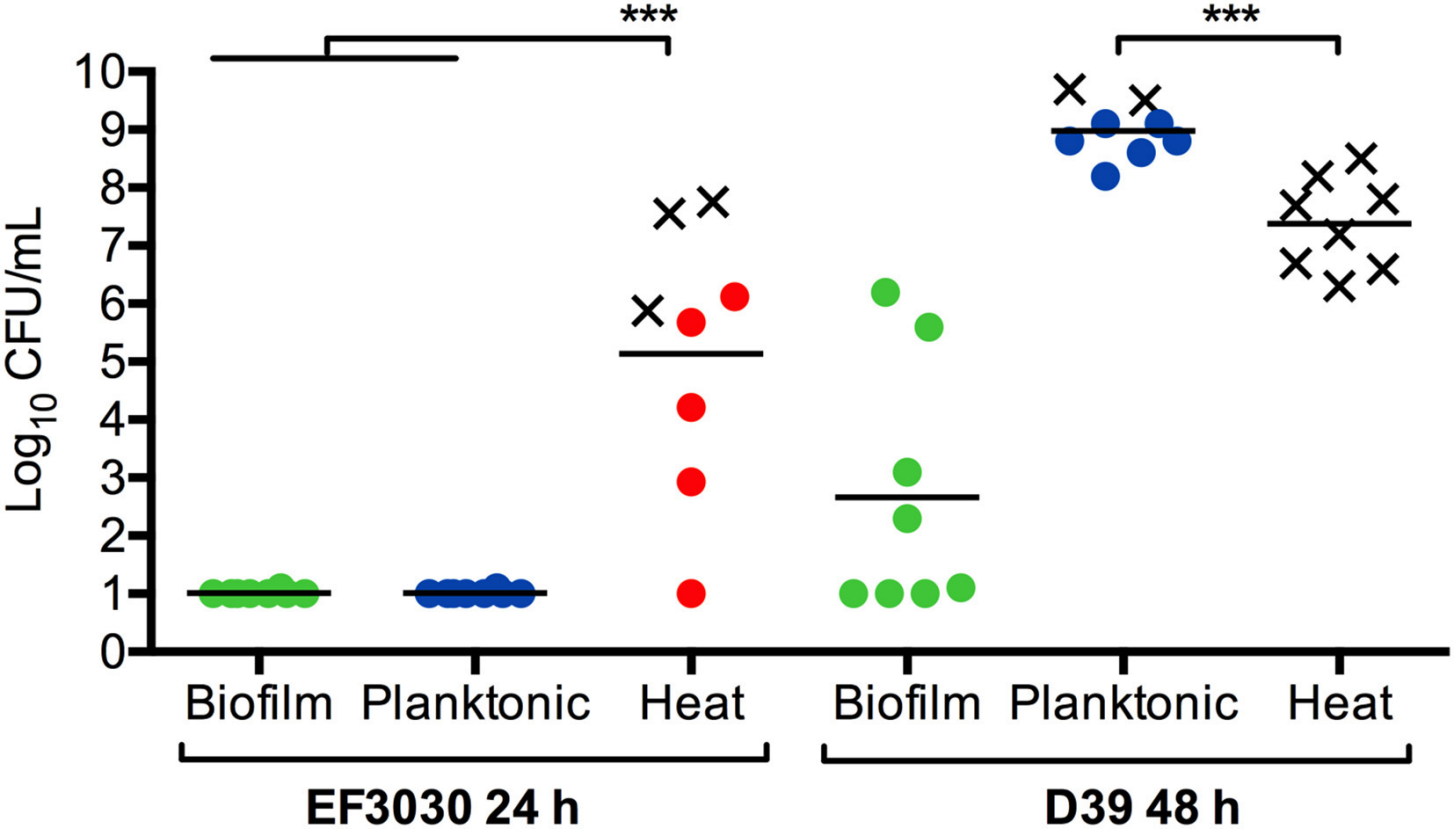
**Biofilm dispersion and infection**. Mice were inoculated intraperitoneally with mechanically disrupted biofilm bacteria (Biofilm; green), broth-grown bacteria (Planktonic; blue), or heat-dispersed bacteria (Heat; red). Bacterial burden was measured in the blood after 24 h for EF3030 bacteria or 48 h for D39 bacteria or when mice became moribund. An X represents a mouse that was euthanized before the end of the experiment. ^***^P < 0.001.

These phenotypes were not only specific to tissue infections. Intraperitoneal challenge with EF3030 or D39 biofilm bacteria resulted in rapid clearance of the bacteria from the bloodstream. D39 bacteria are well-characterized for their invasive potential (Smith et al., 2002) and resulted in a higher bacterial titer in the blood after 24 h. Actively-dispersed D39 bacteria showed an even more aggressive phenotype where the mice were more symptomatic and the majority of the mice had to be euthanized before 24 h based on becoming moribund (Figure 4). The phenotype was especially interesting when the strain EF3030 was used, as this strain when grown in broth failed to induce bacteremia and direct injection of 10^8^ CFUs of EF3030 broth-grown bacteria were cleared within the 24 h. In contrast, injection of approximately 10^5^ CFUs of EF3030 bacteria dispersed from biofilms after IAV infection or exposure to heat or extracellular ATP resulted in septicemia in all mice, with some mice becoming moribund before the end of the experiment (Figure 4). Interestingly, dispersed organisms caused a higher level of inflammation in the bloodstream and the animals became moribund at significantly lower bacterial levels in the blood. These results indicate that actively-dispersed pneumococci, that show high virulence and inflammatory potential, produce infection in animals that more closely resembles invasive pneumococcal disease in humans (Marks et al., 2013). Actively-dispersed pneumococci may thus provide a model that better mimics the physiological phenotype during human infection.

### Dispersed bacteria are a distinct population different from biofilm or planktonic, broth-grown bacteria

The major differences in the virulence phenotype of biofilm bacteria, broth-grown bacteria, and actively-dispersed pneumococci suggest that these populations are distinct and likely have major differences in their transcriptional profiles. Previous studies have shown that some virulence genes are down-regulated in biofilm bacteria compared to broth-grown bacteria (Sanchez et al., 2011b) and that changes in the host environment results in alterations of pneumococcal transcriptional profiles (Orihuela et al., 2004b; Ogunniyi et al., 2012). These environmental signals include IAV-induced host responses such as rises in temperature (fever), nutrient availability, ion concentrations, and proinflammatory cytokines (Bakaletz, 2010; Grebe et al., 2010; Weiser, 2010) that trigger biofilm dispersal, leading to a distinct population of biofilm-dispersed bacteria showing an increased ability to disseminate and cause disease (Marks et al., 2013).

### Transcriptional differences in actively-dispersed pneumococci

To better understand the transcriptional profiles of dispersed bacteria, gene expression profiles of dispersed bacteria were compared to expression profiles in planktonic, broth-grown bacteria and to biofilm bacteria grown on fixed or live epithelial cells for 48 h using qRT-PCR of selected genes (Marks et al., 2013). Similar to previous work, competence genes were up-regulated in biofilm-grown bacteria compared to dispersed and planktonic, broth-grown populations while other genes involved in virulence, such as *cps* (capsule), *ply* (pneumolysin), *pavA* (adhesin), and *licD2* (opaque phenotype), were down-regulated during biofilm growth, consistent with previous reports (Sanchez et al., 2011b; Marks et al., 2012b). More importantly, planktonic, broth-grown bacteria had different expression of *lytA, licD2*, and *pavA* compared with dispersed bacteria, the latter population showing significantly higher expression. These differences in gene expression suggests that the three populations are phenotypically distinct. Besides virulence differences, actively-dispersed pneumococci had a higher opaque to transparent ratio, adhered poorly to HRECs, but invaded and killed HRECs more effectively, as well as induced higher levels pro-inflammatory cytokine responses from the exposed HRECs (Marks et al., 2013).

In addition to the gene targeted RT-PCR approach, we used RNA-seq to obtain a global transcriptional profile among different samples, and identified complex alterations in the pneumococcal transcriptome in response to IAV-induced changes in the environment (Pettigrew et al., 2014). Among the actively-dispersed pneumococcal populations, IAV-induced dispersion had the most impact on the pneumococcal transcriptome compared to biofilm-grown bacteria. This was seen in both the fold-change and the number of differentially regulated genes. When combining the changes observed in IAV-, heat-, and ATP-dispersed populations of pneumococci, 90 differentially regulated genes were significantly changed in the same direction in at least two out of three dispersed populations compared to biofilm-grown bacteria. In general, carbohydrate metabolism, stress response, and known virulence factors were up-regulated in dispersed populations while genes associated with competence, amino acid metabolism, pyrimidine and purine metabolism, translation, and some regulatory genes were downregulated. These data correlate very well with a recent study demonstrating an increased expression of genes involved in cell wall biosynthesis, translation, and purine and pyrimidine metabolism in biofilm bacteria (Yadav et al., 2012). The data also correlate in part with a recent proteomics analysis that showed a changed metabolism in biofilm bacteria (Allan et al., 2014). However, as this analysis compared biofilms to planktonic, broth-grown bacteria that are very different in their transcriptional profile to actively dispersed bacteria, a direct comparison of the results are difficult to make.

Among the 20 out of 90 genes that were regulated in different directions in the dispersed populations, eight were genes involved in bacteriocin production and secretion. These genes were upregulated in IAV- and heat-dispersed pneumococci that showed the highest virulence in our murine model and were down-regulated in the ATP-dispersed population that showed the least virulent phenotype, suggesting a potential role of bacteriocins in virulence. Overall, similar patterns were seen in the comparison between actively-dispersed and planktonic, broth-grown bacteria.

The RNA-seq data showing differentially expressed genes involved in carbohydrate metabolism corresponded well with the direct measurement of glucose metabolism among the pneumococcal populations, with a higher production of intracellular ATP and lactate secretion (main product of pneumococcal glucose fermentation) in dispersed populations compared to biofilm-grown bacteria. In addition, biofilm bacteria had a lower baseline ATP level, suggesting low metabolic activity. Genes regulating carbohydrate metabolism have been associated with tissue-specific disease (Orihuela et al., 2004a; Iyer and Camilli, 2007; Ogunniyi et al., 2012), which is a similar pattern seen with the more virulent dispersed populations showing upregulation of genes associated with carbohydrate metabolism. However, there was not a direct correlation between glucose metabolism and virulence among the pneumococcal populations. In addition, there was variability in gene regulation and glucose metabolism among heat- and ATP-dispersed populations even though heat-dispersed pneumococci were more similar though not as virulent as IAV-dispersed while ATP-dispersed pneumococci were the least virulent dispersed population. These data indicate that virulence and transcriptional changes in response to environmental signals are complex.

## Conclusions

**Figure 5:**
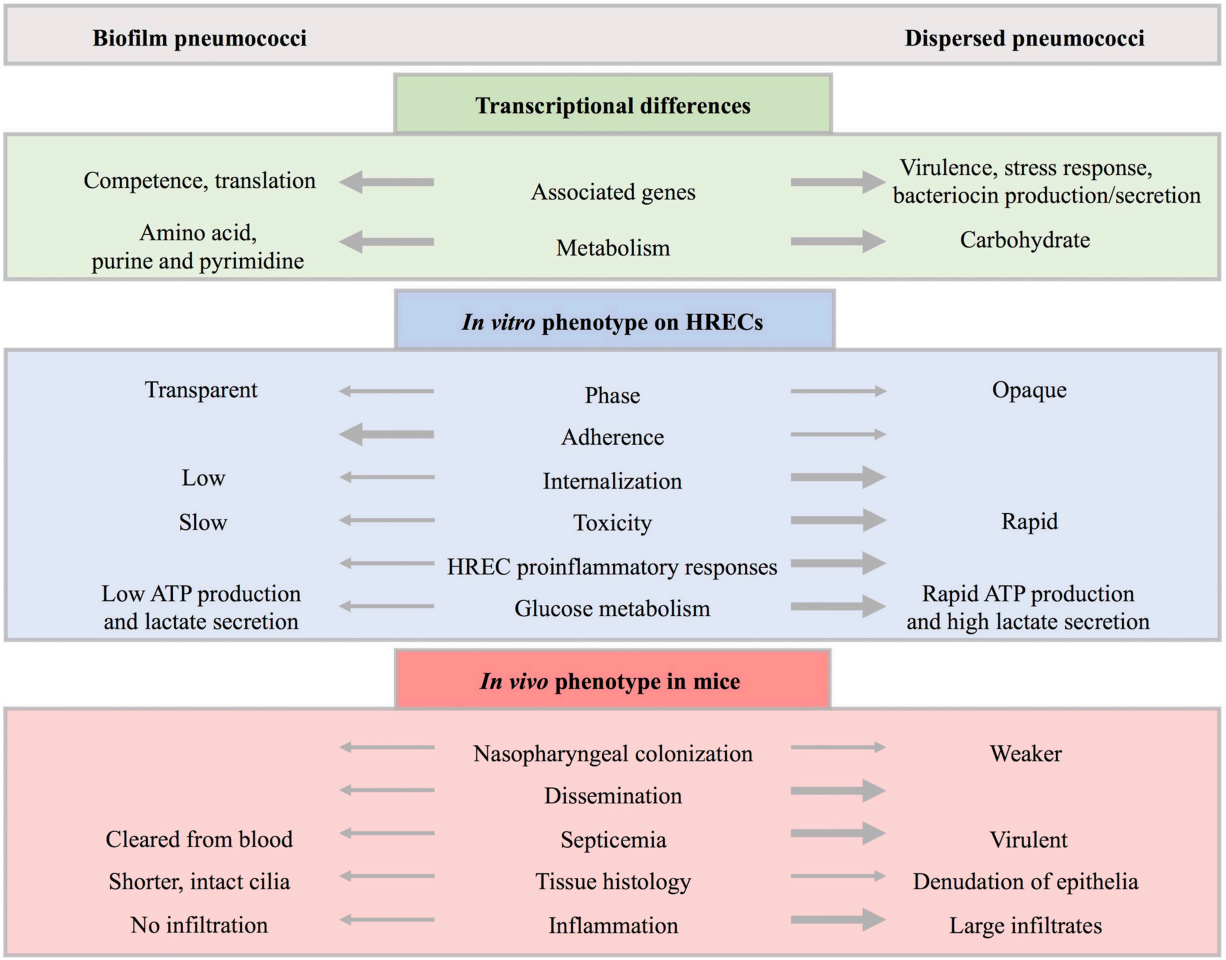
**Comparison of biofilm and dispersed pneumococcal populations**. Biofilm-grown and biofilm-dispersed bacteria are distinct populations with different transcriptional profiles and phenotypic properties. In general, biofilm bacteria upregulate genes associated with competence while dispersed bacteria upregulate genes associated with virulence. Furthermore, genes associated with carbohydrate metabolism, bacteriocin production and secretion, stress response, and virulence factors are upregulated in dispersed populations compared to biofilm-grown bacteria while genes associated with colonization such as competence and fratricide, genes involved in amino acid metabolism, purine and pyrimidine metabolism, and translation are downregulated. This is in agreement with glucose metabolism assays where biofilm bacteria ineffectively produce ATP or secrete lactate in contrast to the rapid metabolism of glucose seen in actively-dispersed populations. In addition, biofilm bacteria are predominantly transparent in contrast to primarily opaque dispersed bacteria with upregulation of capsule expression. *In vitro* studies indicate that biofilm bacteria are less virulent and show increased adherence to human respiratory epithelial cells (HRECs). In contrast, dispersed bacteria are less adherent and have an increased ability to invade and kill HRECs with a higher induction of key cytokines involved in pro-inflammatory responses from exposed HRECs. *In vivo* studies show that both populations are able to colonize the murine nasopharynx, however, dispersed bacteria colonize more weakly and result in dissemination with a significantly higher bacteria load. In the mouse septicemia model, dispersed populations are virulent while biofilm bacteria are quickly cleared from the blood. When comparing infected mouse tissue, biofilm bacteria resulted in shorter, intact cilia with no inflammatory infiltration. This is in contrast to the denudation of epithelia and large inflammatory infiltrates seen in tissue infected with actively-dispersed bacteria.

Colonization by *S. pneumoniae* precedes disease and studies have shown that colonization is a necessary step in pneumococcal pathogenesis (Weiser, 2010; Simell et al., 2012). While there is evidence for the role of biofilms in disease (Hall-Stoodley et al., 2006; Sanderson et al., 2006; Hoa et al., 2009; Reid et al., 2009; Sanchez et al., 2010; Weimer et al., 2010; Sanchez et al., 2011b; Trappetti et al., 2011b; Blanchette-Cain et al., 2013), the role of biofilms in pneumococcal colonization has only recently been investigated. Asymptomatic colonization occurs within complex multicellular biofilm communities (Munoz-Elias et al., 2008; Marks et al., 2012a) while pneumococci from the blood and sputum exist as diplococci (Tomasz et al., 1964). Host–bacterial interactions are necessary for optimal biofilm formation displaying increased antibiotic resistance (Marks et al., 2012a). Furthermore, environmental conditions in this niche are important for increased genetic exchange and increased fitness either by expanding the genes available or through protective effects (Marks et al., 2012b). As summarized in Figure 5, these sessile, predominately transparent phase communities down-regulate virulence factors and show increased adherence, low invasiveness and toxicity to HRECs, and elicit low cytokine responses (Marks et al., 2013). Biofilm bacteria found during colonization are avirulent, but are a source of pathogenic bacteria upon signals from IAV-induced changes in the environment (Marks et al., 2013).

Respiratory viruses trigger host responses and signals resulting in changes in the niche environment, including nutrient availability, temperature, and ion concentration that play an important role in the pneumococcal transition from commensal bacteria to disease-causing pathogen (Marks et al., 2013) (see Figure 4). Furthermore, actively-dispersed pneumococci have distinct transcriptional profiles compared to biofilm or planktonic, broth-grown bacteria, showing upregulation of carbohydrate metabolism and bacteriocin production and down-regulation of genes associated with competence, amino acid metabolism, purine and pyrimidine metabolism, and other regulatory genes (Pettigrew et al., 2014). Dispersion may be an important survival strategy as exposure of asymptomatically colonized mice with host responses induced dissemination of pneumococci into the lungs and middle ear. Recognition of these host responses suggests that inter-kingdom signaling in an important mechanism of transition from asymptomatic colonizer to pathogen.

### Future studies

Our recently developed biofilm models have been instrumental in increasing our knowledge regarding pneumococcal colonization of the nasopharynx and the transition to invasive disease. Further understanding of pneumococcal biofilm formation will be important for addressing the spread of antibiotic resistance, serotype switching, vaccine escape, and protective effects in the context of co-colonization. In addition, biofilms have induced competence and capsule downregulation associated with increased transformation, which may be important for future models studying genetic exchange.

IAV-induced responses triggered the dispersion of a distinct population of pneumococci, suggesting that pneumococci recognize inter-kingdom signals. This model of pathogenesis with co-infection of IAV and pneumococci may be adapted to model co-infection and the transition to disease for other upper respiratory tract commensals that also experience increased virulence after IAV infection (e.g., *Staphylococcus aureus*). In addition, our model could also be used to study a wider range of pneumococcal strains, such as clinical isolates that are not currently virulent in mouse models.

Future studies capitalizing on the RNA-seq data should focus on the role of carbohydrate metabolism, bacteriocin receptors, and other genes encoding surface proteins upregulated during invasive disease as these may represent novel targets for developing therapeutics. The transcriptional differences found between the pneumococcal populations explain the differences in virulence, however, future goals will involve understanding the mechanism involved in the induction of disease.

### Conflict of interest statement

The authors declare that the research was conducted in the absence of any commercial or financial relationships that could be construed as a potential conflict of interest.
